# Precise Management of Chiari Malformation with Type I

**DOI:** 10.3389/fsurg.2022.850879

**Published:** 2022-03-28

**Authors:** Fuyou Guo, Mehmet Turgut

**Affiliations:** ^1^ Department of Neurosurgery, The First Affiliated Hospital of Zhengzhou University, Zhengzhou, China; ^2^ Henan International Joint Laboratory of Nervous System Malformation, Zhengzhou, China; ^3^ Department of Neurosurgery, Aydın Adnan Menderes University Faculty of Medicine, Efeler, Aydın, Turkey; ^4^ Department of Histology and Embryology, Aydın Adnan Menderes University Health Sciences Institute, Efeler, Aydın, Turkey

**Keywords:** algorithm, chiari malformation, imaging, management, surgery

## Abstract

Diagnosis of Chirai malformation type I (CM-I) is based on magnetic resonance imaging of the brain or cervical spinal cord. The main goal of surgery is to relieve the blockage to the free pulsatile flow of cerebrospinal fluid beyond the foramen magnum and to stop the progression of a syringomyelia. Despite recent advances in imaging and surgery, even today, there is no consensus on optimal management of CM-I. Ongoing focus is devoted to a better consideration of the pathophysiology of CM-I and the development of more effective medical and surgical treatments. It is hoped that proposed algorithm helps the neurosurgeon to provide a precise management for patients with CM-I in advance.

## Introduction

The Chiari malformation type I (CM-I) is described as the downward displacement of cerebellar tonsils through the foramen magnum into the upper cervical spinal canal. Numerous studies across the CM is riddled with questions, but hardly any definite answers have come out even though the improvement of neuroimaging examination and basic research. Just as Rebecca Voelker advocated (1): How and why does CM develop? Which decision making criteria should be used to classify the cases as mild, moderate, or severe? Which patients benefit from surgical treatment? All the questions still remained to be eludicated in 21^st^ century. Although the standard treatment for symptomatic CM-I is foramen magnum decompression (FMD) to relieve pressure on the medulla and facilitate cerebrospinal fluid (CSF) flow, however, there is not any current guideline or consensus on management for CMs (2, 3). In order to avoid the potentially surgical failure following the decompression of posterior cranial fossa (PCF) (also known as FMD) in clinical practice, it is important to accurate evaluation through multiple tools before surgery and subsequently to perform an individual operation. Hence, the essential strategy focused on CM-I surgery during perioperative period were highlighted in this article.

### Multi-Modality Neuroimaging Precise Assessment

**Figure 1:**
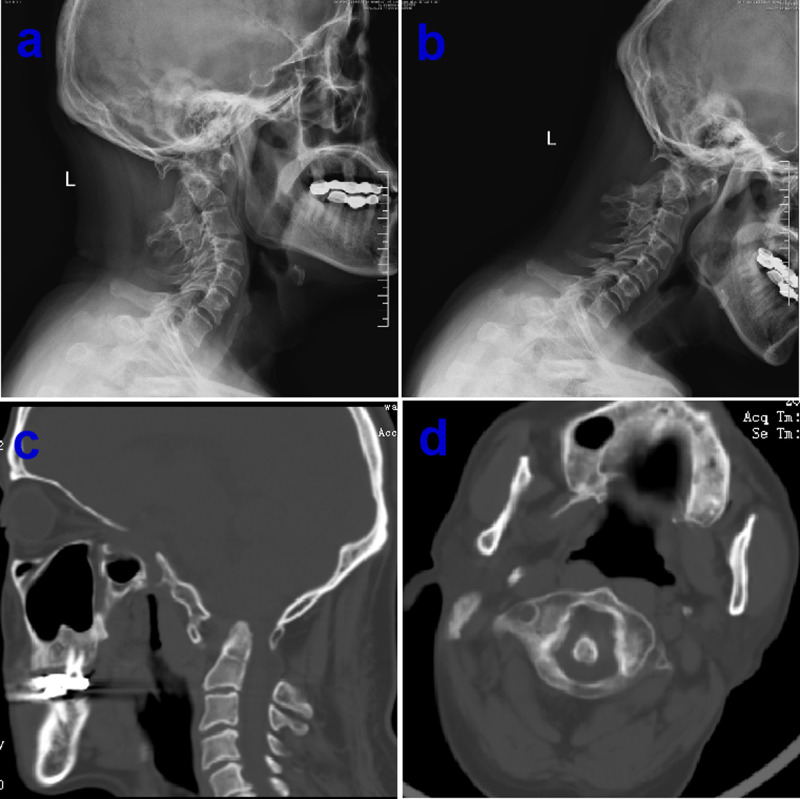
Preoperative over-extension/flexion dynamic X-ray showing normal bone structure of atlantoaxial dislocation, basilar invagination and assimilation of atlas (**A** and **B**), computerized tomography (CT) examination confirm concomitant multiple bony abnormalities in the same patient (**C** and **D**).

**Figure 2:**
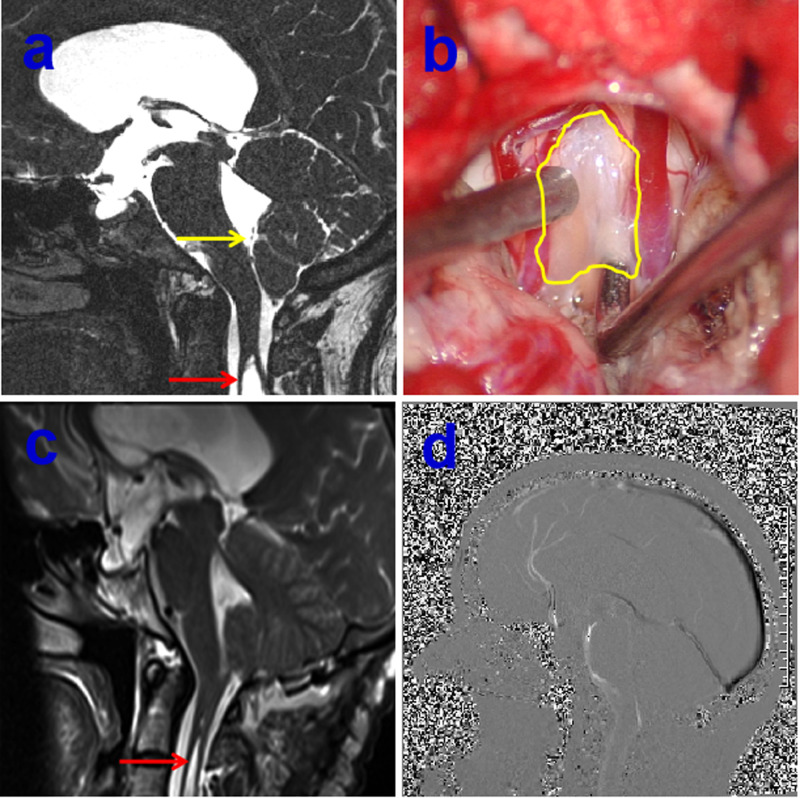
Preoperative 3-dimensional T2-weighted CISS sequence demonstrating arachnoid veil resulting in the cerebrospinal fluid (CSF) blockage, yellow arrow indicating the arachnoid veil, red arrow showing the syringomyelia (**A**), intraoperative intradural exposure of the craniocervical junction dorsally illustrating an arachnoid veil causing fourth ventricular outlet obstruction, yellow circle showing arachnoid veil (**B**), postoperative MRI showing the remove of arachnoid vei and shrinkage of syringomyelia on red arrow (**C**), postoperative sagittal cine-PC showing the improvement of CSF flow (**D**).

The primary goal for any neuroimaging is to assess the diverse type of CM-I so as to pursuing the precise surgery based on preoperative evaluation. In general, the conventional craniocervical junction dynamic position X-ray, including the hyperextension and hyperflexion of neck, and the three-dimensional (3-D) reconstruction of computerized tomography (CT) as well as CT angiography (CTA) also play a vital role in defining the co-existence such as CM-I with atlantoaxial instability as well as anatomic variation of vertebral artery. Previous reports demonstrated that atlantoaxial subluxation was associated with CM on imaging was 14.47%, and posterior fixation is required in all patients following FMD (4), It should be highlighted that CM-I may be regarded as atlantoaxial instability when it is accompanied with bony abnormalities including atlantoaxial dislocation or basilar invagination with ventral brainstem compression. Otherwise, the serious complications of dyspnea or paralysis from cervical cord compression are easily developed if those patients just underwent single decompression of PCF (5), or patients experienced second operation due to delayed instability occurrence (6). Consequently, the first step is to clarify whether CM-I is associated with atlantoaxial instability from the over- flexion/extension dynamic X-ray or CT examination (Figure 1). There is no doubt that the diagnosis of CM-I is based on magnetic resonance imaging (MRI) of the brain or cervical spinal cord. Standard T1 and T2 sequences can disclose the location of the cerebellar tonsils and their relationships with the foramen magnum without any difficulty, but special sequences such as high-resolution 3-D T2-weighted images including FIESTA and CISS devoted for imaging of the craniocervical junction (7), which could demonstrate further details of CSF spaces in the region and reveal the existence of CSF blockage and its potential causes in some circumstances (Figure 2). Most importantly, more attention has been paid to the CSF flow detection at the level of the foramen magnum by motion-sensitive MRI techniques (mostly cine phase-contrast [cine-PC]) which have been used a guide to management of the patient with CM-I. The best advantage of these techniques lies in the dynamic process study rather than static anatomical properties. In addition, the abnormality of CSF flow was linked to the poor prognosis, the presence of both ventral and dorsal CSF flow abnormalities on pre-operative MRI was closely relevant with a 2.6-fold reduction in the risk of postoperative recurrence for clinical symptoms (8). Other advanced imaging techniques, which were used for assessment of patients with CM-I, as follows: (1) CSF flow imaging at the foramen magnum with cardiac-gated phase-contrast MRI; (2) cerebellar tonsillar pulsatility at the foramen magnum with cardiac-gated cine MRI (9); and (3) diffusion tensor imaging (DTI), the severity of white matter injury on DTI might be useful for evaluating the postoperative outcome (10, 11). In addition, the X-ray of head and entire spine would help ruling out craniosynostosis and scoliosis (12).

### Precise Surgical Strategy Based on Preoperative Individually Evaluation

**Figure 3:**
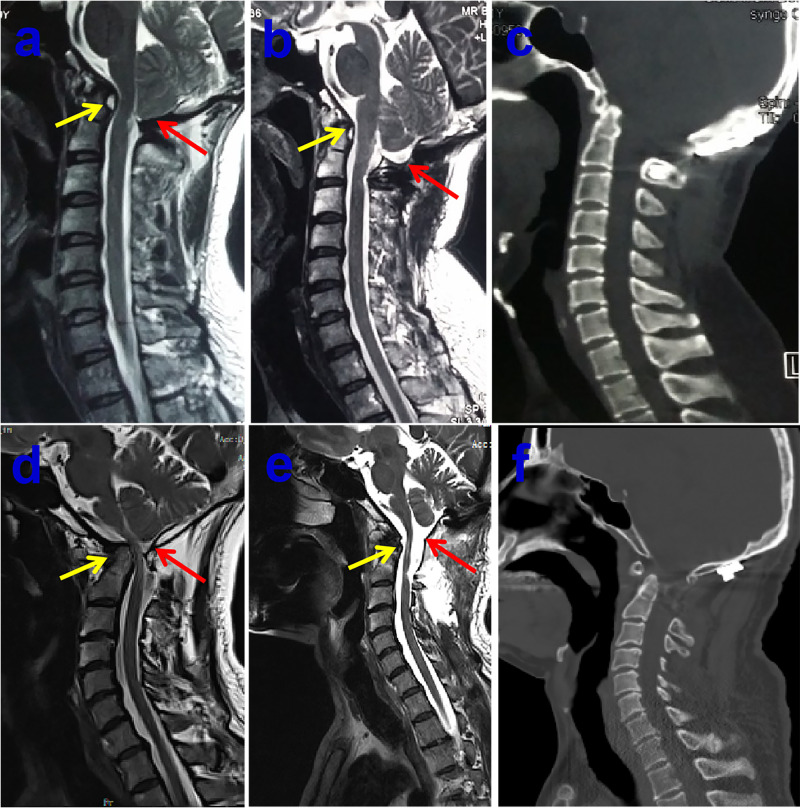
Preoperative midsagittal T2-weighted MRI scan showing slightly ventral compression but no syringomyelia in 40-year-old male patient (**A**), who underwent bony decompression and posterior stabilization of craniocervical junction for numb in both arms and stiff in neck, postoperative midsagittal T2-weighted MRI scan displaying the marked improvement CSF flow anteriorly and posteriorly at the foramen magnum (**B**), the postoperative sagittal CT images showing the posterior reduction and stabilization (**C**). Preoperative midsagittal T2-weighted MRI scan showing seriously ventral compression but no syringomyelia in another 51-year-old male patient, who experienced bony decompression and titanium cage implanting as well as posterior stabilization of craniocervical junction for ataxia and weakness in all limbs (**D**), postoperative midsagittal T2-weighted MRI scan illustrating that the compression of brain stem dissolved excellently (**E**), the postoperative sagittal CT images showing the satisfactory distraction after posterior reduction and stabilization (**F**).

It is our opinion that the correct conception of precise surgery must based on complete assessment of the patient. As a rule, asymptomatic patients who are diagnosed of CM-I without syringomyelia do not frequently benefit from surgical intervention. Satisfactory surgical outcomes were usually obtained in majority of CM-I patients after decompression of PCF. Even today, however, consensus has not been reached as to optimal surgical management for CM-I. The main goal of surgery was relieve the impingement of the tonsilla cerebelli and the blockage to the free pulsatile flow of CSF beyond the foramen magnum. Although there is increasing evidence that only decompression of the bone might be recommended in some CM-I patients due to less invasive surgical procedures, the high failure rate of operation and ineffective in cases with syringomyelia should be pointed out (13, 14), the strategy for optimal management for CM-I should be treated by individually precise assessment. It is wise to remove the arachnoid veil or release the arachnoid adhesive causing fourth ventricular outlet obstruction if preoperative MRI with special sequences of CISS indicates the existence of arachnoid veil/adhesive tissue (Figure 2). Previous literature demonstrated that an arachnoid veil occluding the outlets of fourth ventricle was found in 12% of patients with syringomyelia during operation (15). Hence, the successful surgery was attained only after proper management of arachnoid veil besides opening the both layers of the dura mater. Moreover, there still exists significant controversy on how to deal with the cerebellar tonsils during decompression of PCF. The likelihood of arachnoid scar formation may prevent the neurosurgeon from management aggressively in fear of blood into ventricles. It has been reported that resolution or reduction of the syrinx occurred in about 80–85% of patients after first decompression (10). However, syringomyelia may persist in up to 10–20% of patients, owing to either inadequate decompression and/or excessive fibrotic scar tissue formation which result in impairment of CSF flow (16). On the other hand, patients treated with cautery to the tonsilla cerebelli had 6.11 times greater likelihood of improvement in their syrinx without increased perioperative complications (17), likewisely, decompression of PCF with duraplasty as well as obex exploration was connected with a significantly higher odds of syrinx and resolution of symptoms compared with bone decompression, complications were not notably elevated when any duraplasty or duraplasty with obex exploration relative to only decompression of the bone (18). In addition, the complex Chiari malformation (CCM) characterized usually by abnormal craniocervical bony anatomy such as odontoid retroflexion, occipitalization of the atlas, basilar invagination, or abnormal clival-cervical angle, compared with simple CM, much more attention should be paid for simultaneous fixation except decompression of PCF (**Figure 3**) (19). Otherwise, the serious complication of occipitocervical instability frequently occurred following single decompression of PCF, it is extremely necessary to perform posterior fixation and fusion as well as one-stage decompression when patients with maximal depth of dens to the line from the basion to the inferoposterior portion of the C2 body (pBC2 line) of more than 9 mm and clival-cervical angle or clivoaxial angle (CXA) <125° based previous studies (19–23). In addition, the intraoperative ultrasound could be used to assess the degree of decompression achieved following laminectomy, the flow of CSF in the subarachnoid space and the persistence of syrinx (24).

### Precise Outcome Analysis by Quantitative Measurable Tools

For long time very unspecific patient reported outcome measures (PROMs) were used for CM-I patients, including Patient Global Impression of Change, Odom’s criteria and EQ-5D-5L (25). Whereas the use of CM-I specific tools allowed surgeons to focus on patients’ appreciation of postoperative clinically important differences. The core characteristic of precise management for CM-I should be evaluated accurately based on the quantitative measurable tools. It is well known that description of symptoms is usually subjective due to the lack of a reliable and unified criteria, noticeable bias of clinical outcomes easily occurred in term of patient’s subjective complaint, especially whether operative intervention will virtually result in benefit should be analyzed by reliable quantitative measurable tools rather than qualitative methods such as symptom improvement or deterioration. Hence, Aliaga et al. (26) firstly reported that a novel scoring system for assessing postoperative outcome of CM-I in 2012, which was proved that this scoring system is useful and reliable by quantitatively assessment according to the Chicago Chiari Outcome Scale (CCOS) (27–31). Another role model of a prognostic scoring system for outcome of patients with CM-I is the Chiari Severity Index (CSI) which was put forward by Greenberg et al. (32). Subsequently, Seow Operative Score was applied for decision-making intervention for pediatric CM patients in 2019 (33). In addition, the recent novel score termed SHORE was advocated by Feghali et al. (34), which is a new predictive tool for improvement in adult CM-I after decompression surgery. More and more evidences demonstrated that variety of quantitative measurable tools were developed and used for individually evaluation, which eventually lead to precise outcome analysis for patient with CM-I.

### The Algorithm of Precise Management for CM-I

**Figure 4:**
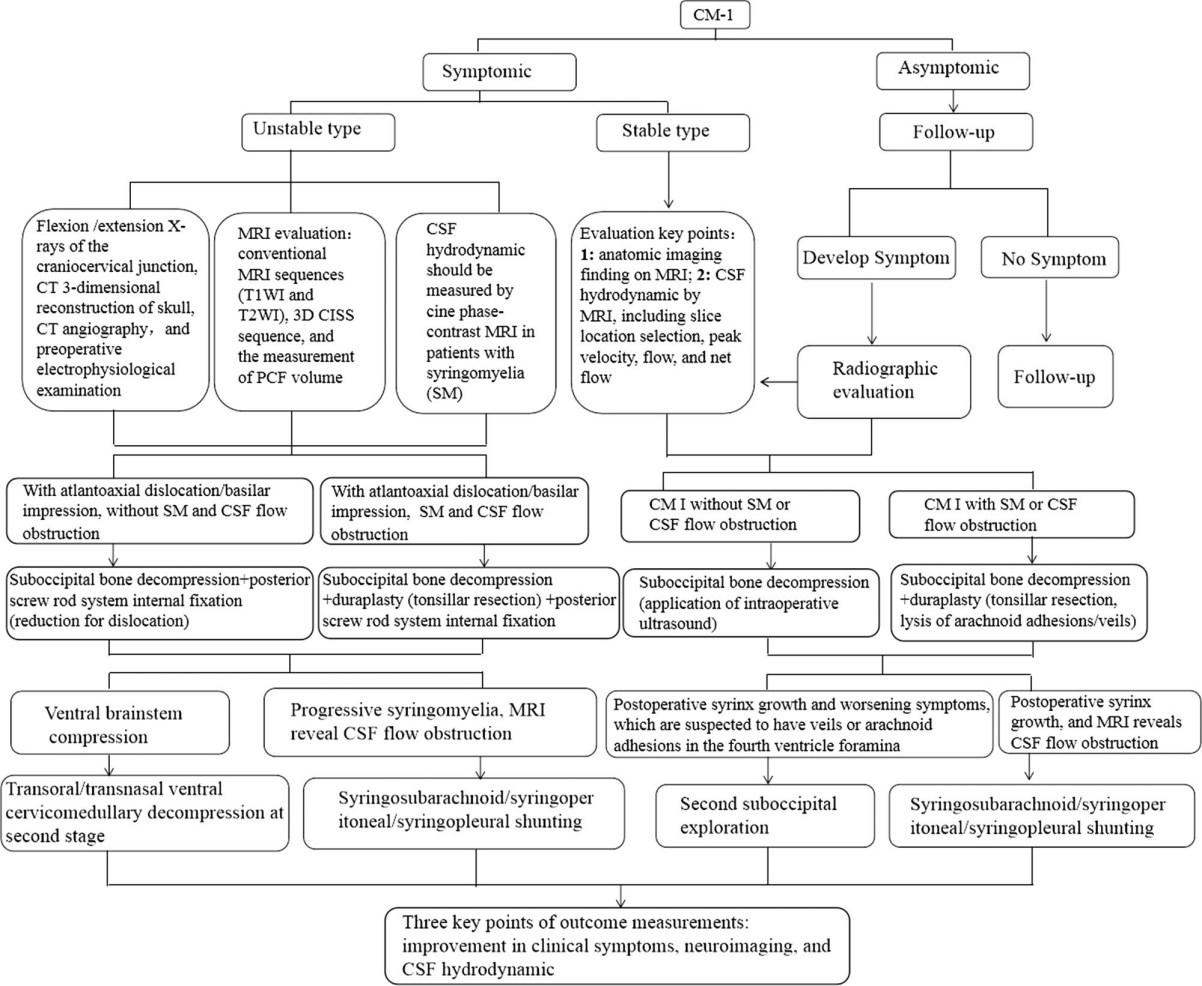
Algorithm flowchart.

There has been much controversy about the optimal treatment strategies for CM-I, moreover, there are insufficient and conflicting evidences regarding the outcome of surgical decompression. Surgery is routinely recommended for patients with significant symptoms or to stop the progression of a syringomyelia. Discrete types of surgical decompression procedures with different invasivenes, ranging from from only decompression of bony and ligamentous structures to coagulation of tonsilla cerebelli, have been suggested in the management of such patients. We strongly believe that the procedure of management choice must be modified on the individual patient according to viewpoint of Balestrino et al. (35). In the light of our institutional experience, we described an algorithm for precise management of CM-I, including the preoperative multi-modality neuroimaging and individual surgical therapy, as shown in **Figure 4**. Ongoing focus is devoted to a better consideration of the pathophysiology of CM-I and the development of more effective medical and surgical treatments.
